# A Fatal Case of Large Cell Neuroendocrine Lung Cancer Metastatic to the Brain: A Case Report

**DOI:** 10.7759/cureus.4728

**Published:** 2019-05-23

**Authors:** Katherine Garcia de de Jesus, Sorab Gupta, Oscar Cisneros, Md. Rezaul Hoque, Ivette Vigoda

**Affiliations:** 1 Internal Medicine, St. Barnabas Hospital Health System / Albert Einstein College of Medicine, Bronx, USA; 2 Oncology, St. Barnabas Hospital Health System / Albert Einstein College of Medicine, Bronx, USA

**Keywords:** large cell neuroendocrine carcinoma, brain metastasis, palliative radiation

## Abstract

Large cell neuroendocrine carcinoma (LCNEC) is part of lung neuroendocrine tumors. LCNEC represents an extremely rare entity with aggressive behavior and poor prognosis. Primary surgery is the mainstream of treatment, although it is rarely amenable due to local or systemic tumor metastasis at the time of the diagnosis. We present a case report of a female patient diagnosed with large cell neuroendocrine lung cancer metastatic to the brain. Noting the low incidence of the disease, the lack of relevant clinical data has resulted in a challenge in diagnosis and management.

## Introduction

Lung neuroendocrine tumors are a group of pulmonary malignancies that originate from neuroendocrine cells and represent around 20% of all lung neoformations. Belonging to that group, small cell lung cancer (SCLC) and large cell neuroendocrine carcinoma (LCNEC) denote the subtypes with the highest grade and aggressive behavior. Both are found more frequently in males in their sixth decade. LCNEC, introduced in 1991 by Travis et al., denotes an extremely infrequent disease, accounting for about 3% of the lung cancers [1]. SCLC and LCNEC share similar clinical and histological behavior, expressing analogous altered genes, such as TP53, KRAS, and RB1 [2]. In the same way, LCNEC exhibits biological features mimicking non-small cell lung cancer (NSCLC), which is conflicting at the time of establishing a treatment [3]. The major risk factor associated with lung neuroendocrine tumors is smoking. Symptoms reported by patients include cough, hemoptysis, chest pain, dyspnea, and night sweats although most of the patients present with an asymptomatic nodule. The distinction between LCNEC and SCLC may be challenging in most cases. The standard of care is surgical resection, when amenable, followed by adjuvant chemotherapy, although the rarity of the disease has not allowed the validation of clinical trials in this setting; thus, optimal treatment guidelines remain indefinite [1].

## Case presentation

A 63-year-old Hispanic female, with a past medical history of chronic obstructive pulmonary disease (COPD) and a 60 pack-year smoking history, presented to the emergency department (ED) complaining of new-onset generalized weakness for the past three days with associated frontal headaches. The remaining review of systems was reported as negative and she was vitally stable.

**Figure 1:**
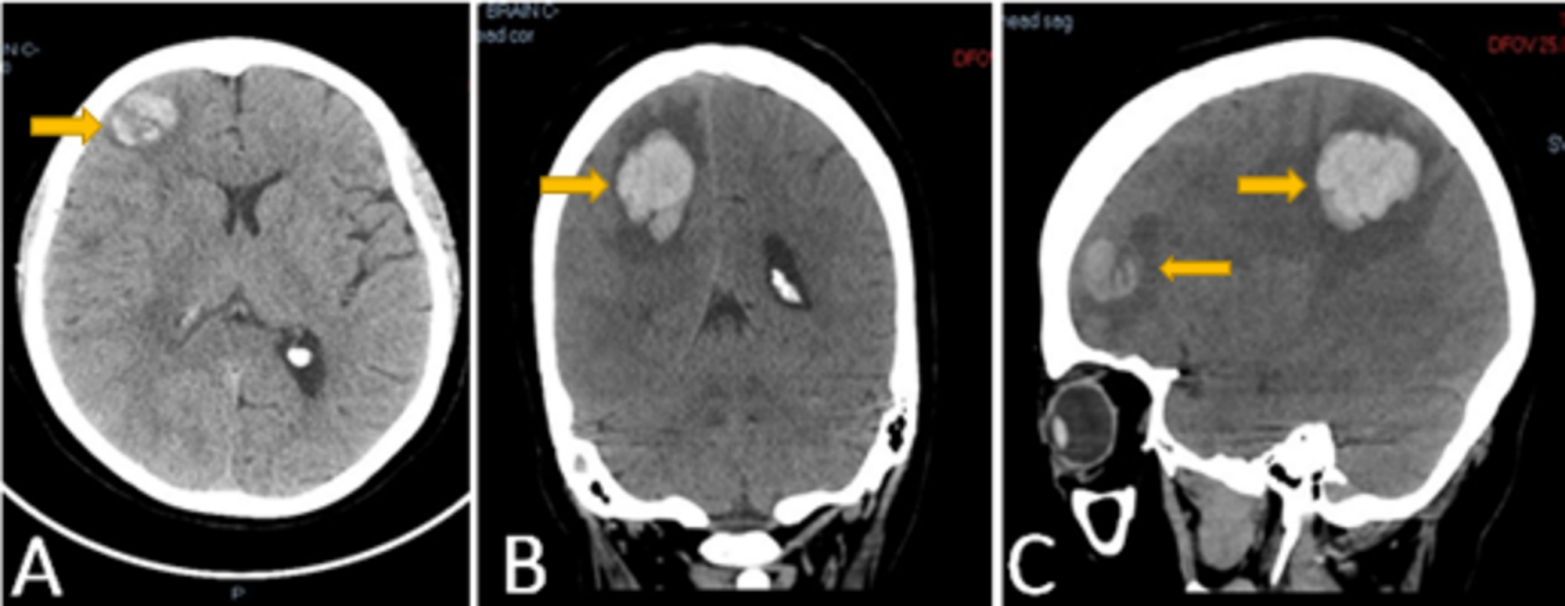
Computed tomography (CT) of the brain Figure A (axial) and B (coronal) views represent a 2.2 cm right frontal lobe area of intraparenchymal hemorrhage with surrounding edema and mild mass effect. Figure C shows a sagittal view, revealing a right parietal lobe, large, 3.7 x 2.5 cm, hyperdense; and a smaller area within the right frontal lobe with surrounding vasogenic edema and possible related underlying mass.

**Figure 2:**
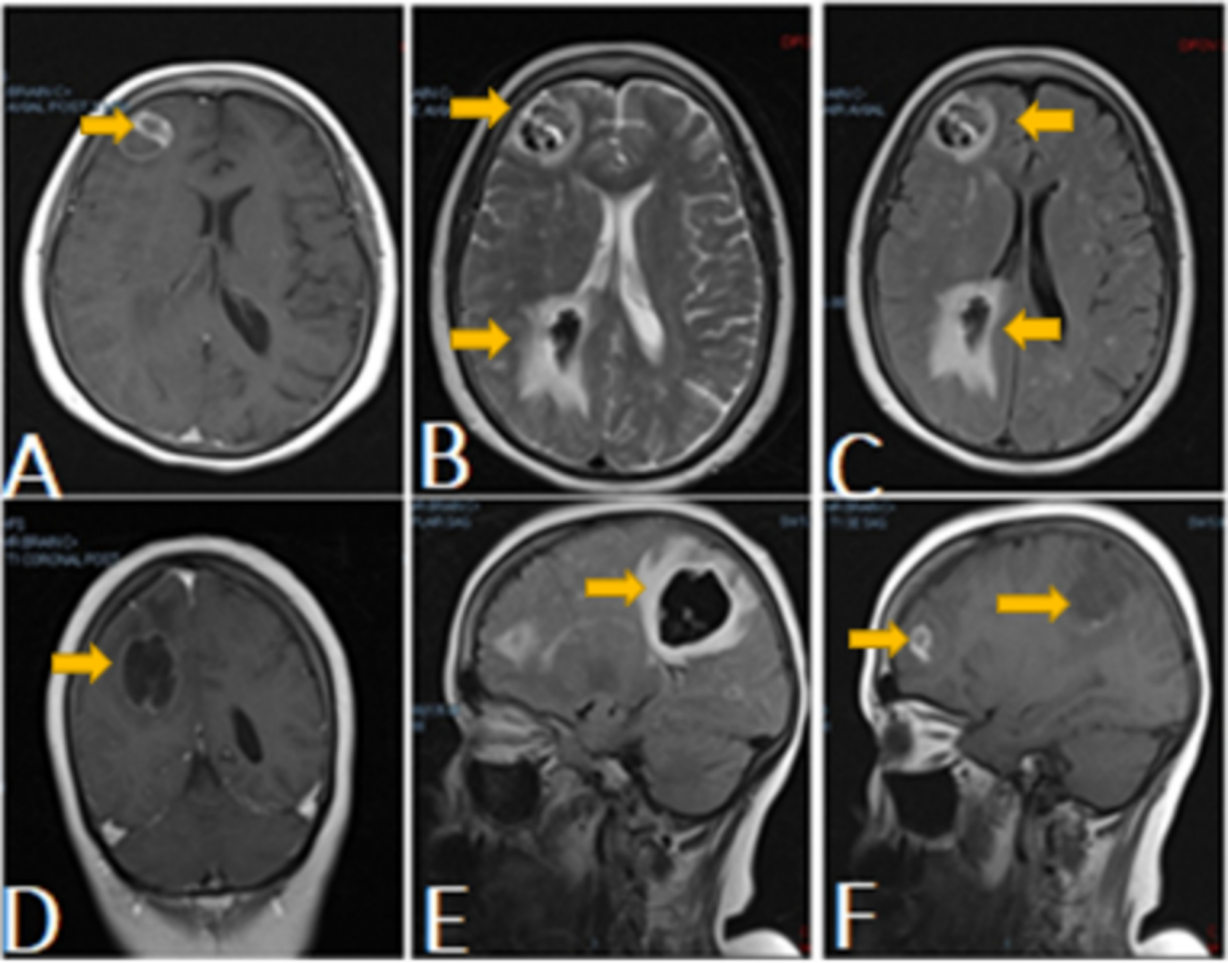
Magnetic resonance imaging (MRI) of the brain MRI of the brain (Figures A-C, axial view) revealed a peripherally enhancing right posterior parietal hematoma with central hypointensity and surrounding edema, along with a septated rim-enhancing focus about the right frontal lobe. Figure D represents the coronal view. Figures E-F (sagittal view) presented a redemonstration of right frontal and posterior parietal intraparenchymal hematomas.

**Figure 3:**
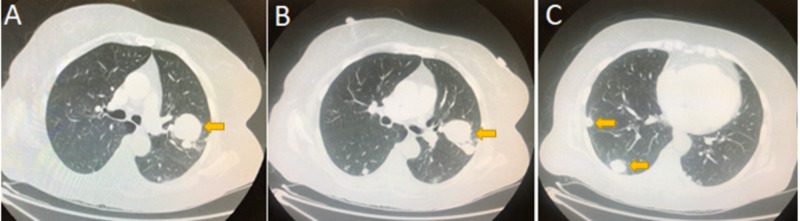
Computed tomography (CT) of the lungs Figure A: CT of the lungs demonstrated a left upper lobe 3.6 cm x 4 cm x 3.7 cm mass with adjacent smaller masses. Figures B-C redemonstrated the left mass and revealed multiple smaller nodules in the right lower, right middle, and right upper lungs.

She was alert and cooperative. Her cranial nerves were intact. Her motor exam, however, was abnormal, with motor strength in the left upper extremity (LUE) being 4/5. The strength and tone of the other extremities were normal throughout. Deep tendon reflexes were decreased bilaterally, but her gait could not be evaluated. Her sensory function was decreased to pin sensation at the LUE and bilateral lower extremities (LEs). Normal sensation was noted in the right upper extremity and face. Laboratory testing was normal. Computer tomography (CT) of the brain was done, revealing a large area of intraparenchymal hemorrhage within the right parietal lobe and a smaller area within the right frontal lobe with surrounding vasogenic edema and a mild rightward midline shift, suggestive of an underlying neoplastic lesion, as can be seen in Figure 1. Brain magnetic resonance imaging (MRI) of the brain delineated a right parietal lobe rim-enhancing lesion measuring up to 39 x 27 mm and a right frontal lobe rim-enhancing lesion with increased enhancement at the anterior aspect measuring up to 21 x 17 mm, as observed in Figure 2. Computed tomography (CT) of the chest with contrast demonstrated multiple pulmonary masses with a dominant left upper lobe of 4 cm mass, as well as a complex septate cystic 2.5 cm mass in the right kidney, as can be observed in Figure 3.

In order to improve the patient's clinical status and obtain an accurate diagnosis, the patient underwent right frontoparietal craniotomy and resection of the hemorrhagic brain lesion. Pathology was amended as poorly differentiated non-small cell type carcinoma, favoring a large cell neuroendocrine carcinoma. The Ki67 proliferation index of the tumor was approximately 50%-80%. Immunohistochemistry was performed, resulting as focally positive synaptophysin, CK7, CK20, CK5, CDX-2, p40, p63, EMA, CD3, CD20, CD45, ER, PR, SOX-10, GATA-3, TTF-1, Napsin, NUT-1, and GFAP. A lung biopsy was performed, revealing poorly differentiated, non-small cell type carcinoma with extensive necrosis and histologic features similar to that of the brain biopsy. The patient was diagnosed as stage IV large cell lung neuroendocrine malignancy. The patient was offered to start brain palliative radiation, for which she endured the initial radiation planning session. During the course of hospitalization, diagnostic nephrectomy was planned for when the patient was more stable; however, the clinical course was complicated by acute respiratory failure secondary to hospital-acquired pneumonia. In the following days, the patient had rapid clinical deterioration. The family’s decision was to transfer the patient to hospice care, where she died a few days later.

## Discussion

Large cell neuroendocrine carcinoma (LCNEC) represents a very rare and hostile malignancy, accounting roughly for 3% of all lung malignancies. This tumor shares clinical and neuroendocrine markers with SCLC, both carrying an unfortunate survival rate. Both are more common in males with a high smoking background in their sixth decade of life. This tumor carries a high mitotic rate. It is composed of large cells with abundant cytoplasm and necrotic component, which helps to differ from SCLC, which has a low nuclear/cytoplasm ratio [1-2].

The disease can be suspected initially by a chest radiograph, but a computed tomography (CT) scan is usually required to delineate the disease, presenting peripherally with irregular margins in most of the cases. Small lung biopsies are habitually nondiagnostic. Pathological diagnosis is challenging in LCNEC. As in our case, in most cases, surgery and subsequent immunohistochemical staining are needed to obtain an accurate diagnosis. Immunohistochemical identify the neuroendocrine markers to validate the diagnosis such as somatostatin, synaptophysin, and chromogranin. Some studies have shown the utility of somatostatin receptor scintigraphy to detect somatostatin receptors (SSTR), which are present in roughly 68% of neuroendocrine cancers [1].

The rarity of the disease and the small population affected has been a caveat for the development of clinical trials. To date, there is no authenticate treatment approach for LCNEC. Based on the National Comprehensive Cancer Network (NCCN), treatment should be based on NSCLC recommendations [3]. Surgery should be offered in all operable patients. Inopportunely, most patients present with local or systemic dissemination, labeling them as unresectable [1]. Most of the patients who undergo surgical resection will develop a recurrence of the disease and those that do not develop recurrent disease run the risk to develop simultaneous second primary cancers [4]. Our patient presented also with a kidney mass suggestive of metastatic disease, but surgical excision was not possible to rule out a second primary malignancy due to a rapid worsening of clinical status. However, more likely, this also represents metastatic disease.

In cases where surgery is not an option, chemotherapy and radiotherapy should be considered. Differing from SCLC, where systemic treatment is given at any stage, LCNEC patients usually receive chemotherapy in more advanced disease, although no specific approach has shown any significant benefit [5]. For more advanced cases, there have been no clinical trials directed to determine the best approach. Existing recommendations are based on an extrapolation of the chemotherapeutic regimens used in NSCLC and SCLC [4]. Currently, four to six cycles of etoposide plus platinum-based chemotherapy is recommended [5]. Based on the similitude with SCLC, in 2016, Iyoda et at. evaluated, in a prospective clinical study, the benefit of cisplatin-etoposide chemotherapy, which is the standard of care in SCLC, as adjuvant therapy after surgery in patients diagnosed with LCNEC. The results of this trial confirmed the superiority of chemotherapy over the control group. Sun et al. also conducted a trial in advanced LCNEC treated with a similar approach to SCLC, with a response rate of 73% [1]. Multiple studies have tried different approaches to LCNEC, however, reported data has not established firm recommendations for management yet.

The benefit of radiotherapy is still uncertain. Some investigators have recommended its use for locally advanced disease. Biological treatment has been an active topic of investigation in SCLC. Few data are available for LCNEC up till now, but based on the results in other lung cancers, it might be a feasible option for patients diagnosed with LCNEC [1]. An immune checkpoint blockade denotes novel therapy for some malignancies. Anti-programmed cell death-1 (PD-1) antibody has radically changed the management and survival in NSCLC. Expression of programmed cell death-ligand 1 (PD-L1) in neuroendocrine lung cancer is being investigated as a prospective target for therapy. Tsuruoka et al. defined an overall beneficial survival in patients with PD-L1 expression. Many clinical studies have studied the prognostic value of PD-L1 expression in LCNEC; nevertheless, preliminary results have shown an unclear survival benefit [6-7].

LCNEC has a high metastatic potential, especially to the central nervous system, as observed in our patient. Brain metastasis has a poor response to treatment. Gamma knife treatment may be a favorable approach in those cases [8]. Prophylactic cranial irradiation (PCI) has shown a clear role in the survival benefit in SCLC. Arsela et al. demonstrated positive outcomes by the implementation of PCI in patients with LCNEC. Nevertheless, other studies have yielded conflicting results. More clinical studies are needed to be conducted to clarify any significant benefit [2].

Within the lung malignancies with non-small histologies, LCNEC is cancer with a worse prognosis, even in its early stages after complete resection [9-10]. Despite a multimodal approach, previous retrospective studies have reported poor survival outcomes. Dresler et at. demonstrated a five-year survival for stage I LCNEC cases of 18% [11]. On the other hand, based on Iyoda et al.'s results, this disease encompasses a five-year survival rate of 35.5% and a five-year disease-free survival rate of 27.4%. The clinical presentation might be very similar to other types of lung cancer, however, histological diagnosis is needed in order to direct more specific therapy.

## Conclusions

Large neuroendocrine lung cancer is a rare malignancy with unique pathological features, extremely aggressive behavior, and poor prognosis. LCNEC biological performance has been reported to mimic SCLC; consequently, patients with LCNEC are often misdiagnosed. Physicians should also be vigilant for the risk of second primary malignancies. Considering the rarity of this disease and the controversies over therapy nowadays, this disease represents a challenge in diagnosis and management. Clinical trials targeting this particular, aggressive cancer are needed to guide more specific management.
